# A Nanoconjugate Apaf-1 Inhibitor Protects Mesothelial Cells from Cytokine-Induced Injury

**DOI:** 10.1371/journal.pone.0006634

**Published:** 2009-08-13

**Authors:** Beatriz Santamaría, Alberto Benito-Martin, Alvaro Conrado Ucero, Luiz Stark Aroeira, Ana Reyero, María Jesús Vicent, Mar Orzáez, Angel Celdrán, Jaime Esteban, Rafael Selgas, Marta Ruíz-Ortega, Manuel López Cabrera, Jesús Egido, Enrique Pérez-Payá, Alberto Ortiz

**Affiliations:** 1 Dialysis Unit, Fundación Jiménez Díaz, Universidad Autónoma de Madrid, Instituto Reina Sofía de Investigación Nefrológica, Madrid, Spain; 2 Servicio de Nefrología, Hospital Universitario La Paz, Madrid, Spain; 3 Polymer Therapeutics Laboratory, Department of Medicinal Chemistry, Centro de Investigación Príncipe Felipe, Valencia, Spain; 4 Laboratory of Renal and Vascular Research, Universidad Autónoma de Madrid, Madrid, Spain; 5 Molecular Biology Department, Hospital Universitario de la Princesa, Madrid, Spain; 6 Peptide and Protein Laboratory, Department of Medicinal Chemistry, Centro de Investigación Príncipe Felipe, Valencia, Spain; 7 Instituto de Biomedicina de Valencia CSIC, Valencia, Spain; 8 Servicio de Microbiología, Fundación Jimenez Díaz, Madrid, Spain; National Institutes of Health (NIH)/National Institute of Environmental Health Sciences (NIEHS), United States of America

## Abstract

**Background:**

Inflammation may lead to tissue injury. We have studied the modulation of inflammatory milieu-induced tissue injury, as exemplified by the mesothelium. Peritoneal dialysis is complicated by peritonitis episodes that cause loss of mesothelium. Proinflammatory cytokines are increased in the peritoneal cavity during peritonitis episodes. However there is scarce information on the modulation of cell death by combinations of cytokines and on the therapeutic targets to prevent desmesothelization.

**Methodology:**

Human mesothelial cells were cultured from effluents of stable peritoneal dialysis patients and from omentum of non-dialysis patients. Mesothelial cell death was studied in mice with *S. aureus* peritonitis and in mice injected with tumor necrosis factor alpha and interferon gamma.

Tumor necrosis factor alpha and interferon gamma alone do not induce apoptosis in cultured mesothelial cells. By contrast, the cytokine combination increased the rate of apoptosis 2 to 3-fold over control. Cell death was associated with the activation of caspases and a pancaspase inhibitor prevented apoptosis. Specific caspase-8 and caspase-3 inhibitors were similarly effective. Co-incubation with both cytokines also impaired mesothelial wound healing in an in vitro model. However, inhibition of caspases did not improve wound healing and even impaired the long-term recovery from injury. By contrast, a polymeric nanoconjugate Apaf-1 inhibitor protected from apoptosis and allowed wound healing and long-term recovery. The Apaf-1 inhibitor also protected mesothelial cells from inflammation-induced injury in vivo in mice.

**Conclusion:**

Cooperation between tumor necrosis factor alpha and interferon gamma contributes to mesothelial injury and impairs the regenerative capacity of the monolayer. Caspase inhibition attenuates mesothelial cell apoptosis but does not facilitate regeneration. A drug targeting Apaf-1 allows protection from apoptosis as well as regeneration in the course of inflammation-induced tissue injury.

## Introduction

Tissue injury is an unwanted adverse effect of inflammation. Peritoneal dialysis (PD) is a renal replacement therapy modality that is marred by episodes of bacterial infection, leading to localized inflammation evidenced as peritonitis [1]. PD represents an interesting model of inflammation since the technique consists of and allows repeated non-invasive access to the peritoneal cavity, allowing both monitoring of the inflammatory process as well as therapy by local delivery of drugs. Currently the therapy of peritonitis consists of local intraperitoneal delivery of antibiotics and heparin [2]. One of the main peritoneal manifestations of inflammatory tissue injury is loss of mesothelial cells, which occurs both during chronic PD and in acute inflammatory episodes [3], [4]. Apoptotic mesothelial cells are lost in the peritoneal effluent of stable PD patients and the number of peritoneal effluent apoptotic mesothelial cells increases 80-fold during peritonitis [5]–[7]. Counting effluent apoptotic cells will underestimate apoptosis, since the apoptotic features have a half-life of 1–2 hours and most apoptotic cells are engulfed by phagocytes [8]. Lethal cytokines are among the endogenous mediators that cause mesothelial cell death [5], [6], [9]–[11]. FasL directly promotes mesothelial cell apoptosis [6]. By contrast, neither TNF nor TRAIL alone modulate mesothelial cell survival [6]. However, most extracellular inputs are not processed in isolation, rather, multiple inputs are perceived and integrated by cells in a proinflammatory milieu [12]. In this regard, mesothelial cells are immersed in a complex microenvironment and inflammatory cytokines may cooperate to influence on mesothelial cell fate. Other inflammatory mediators, bacterial infection, tumor cells, PD solutions and asbestos also promote mesothelial cell apoptosis [7], [11], [13]–[18].

Apoptosis is an active model of cell death that regulates cell number [9], [19], [20]. Understanding the regulation of apoptosis has possible therapeutic relevance, since it is regulated by the activation of intracellular lethal molecules in response to the cell microenvironment [9], [19]–[21]. Among them, caspases are a family of intracellular cysteine proteases that behave as activators and effectors of apoptosis, and play a central role in the process [20], [22]. Caspase-8 is the canonical initiator caspase engaged by lethal cytokines that activate cell death receptors. In turn, caspase-8 recruits the mitochondrial pathway for apoptosis and activates executioner caspases, such as caspase-3, that are responsible for cell death. Activation of the mitochondrial pathway, leads to the release of proapoptotic molecules such as cytochrome c into the cytoplasm, which, in the presence of dATP, induce the formation of the Apaf-1 (apoptotic protease activating factor 1)-containing macromolecular complex called the apoptosome. This complex, in turn, binds to and activates caspase-9. Mature caspase-9 activates effector caspases [23]. Caspase inhibitors prevent leukocyte apoptosis induced by conventional, glucose-containing PD solutions [6], [24], [25]. However, recent reports have emphasized non-apoptotic functions of caspases including promotion of cell proliferation that contributes to tissue regeneration [26]–[28]. In addition, in certain epithelial cell types, caspase inhibition may transform a mild proapoptotic response into an intense necrotic response to lethal cytokines [29]. We now explore the cooperation between inflammatory cytokines in modulating human mesothelial cell fate and possible therapeutic interventions to prevent mesothelial cell death during inflammation. In particular we have analyzed its modulation by classical caspase inhibitors as well as by targeting the activity of the apoptosome. Recent reports have proposed the apoptosome as an interesting target for the development of apoptosis inhibitors [30]–[32]. Indeed, our results showed that chemical inhibition of apoptosome activity with a nanoconjugate Apaf-1 inhibitor is effective in protecting mesothelial cells from cytokine-induced apoptosis and, in contrast to caspase inhibition, it allows wound healing and long-term recovery.

## Results

### Regulation of mesothelial cell apoptosis by cooperation of inflammatory cytokines

**Figure 1:**
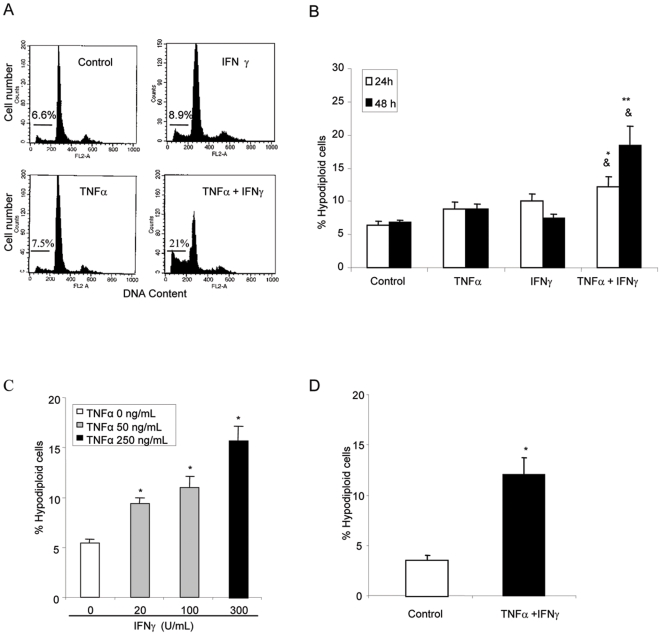
Cooperation of cytokines induces apoptosis in mesothelial cells. A) Representative flow cytometry of DNA content diagrams. Note the increase in the hypodiploid, apoptotic population in HPMC cultured for 48 h in presence of TNFα/IFNγ. B) Quantification of apoptosis in HPMC by flow cytometry. The combination of TNFα/IFNγ induces apoptosis. When not specified, 300 U/mL IFNγ and 250 ng/mL TNFα were used, * p<0.04 vs each individual cytokine at 24 h. ** p<0.001 vs each individual cytokine at 48 h. & p<0.001 vs control. Mean±SEM of 6 different experiments. C) Dose-response in HPMC. Mean±SEM of 4 different experiments * p<0.05 vs control. D) Quantification of apoptosis in HOMC by flow cytometry. The combination TNFα/IFNγ for 48 h induces apoptosis. *p<0.005 vs. control. Mean±SEM of 4 different experiments.

Acute demesothelization occurs during experimental and human peritonitis, and is associated with an increased rate of mesothelial cell apoptosis [3]–[6]. During peritoneal inflammation numerous cytokines are released locally. Among them we find IFNγ and TNFα [33], [34]. Although TNFα is, by itself, a potentially lethal cytokine [19], we did not observe a lethal effect of TNFα in human peritoneal mesothelial cells (HPMC) cultured from the effluent of stable PD patients (**Fig. 1. A, B**). IFNγ increased TNFα lethality (**Fig. 1.A, B**) [6]. The combination of cytokines induced HPMC apoptosis in a time- and dose-dependent manner (**Fig. 1.B, C**). By contrast, cytokines had no effect on the percentage of cells on the S or M phase of the cell cycle, as assessed by flow cytometry of DNA content (range 9 to 10% for the different conditions). The combination of IFNγ and TNFα also induced apoptosis in human omental mesothelial cells (HOMC) cultured from the omentum of non-uremic patients (**Fig. 1.D**).

### Caspases mediate mesothelial cell apoptosis induced by cytokine cooperation

**Figure 2:**
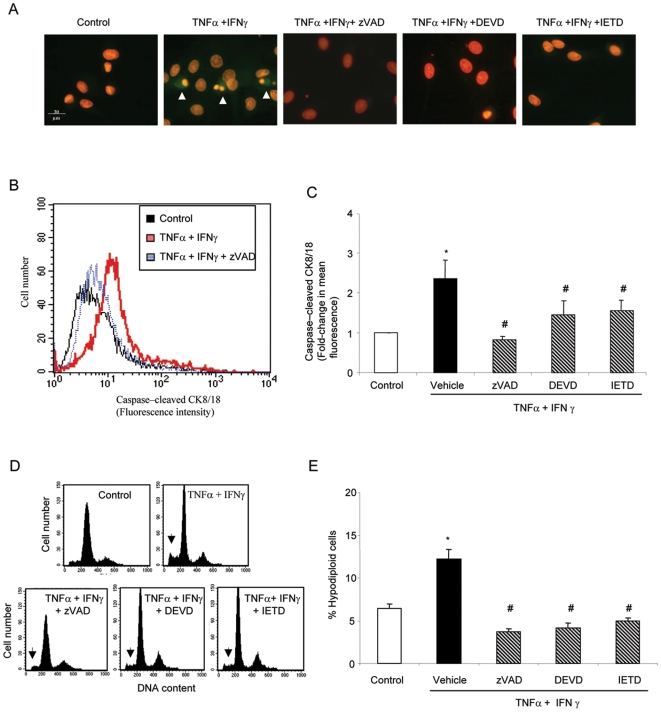
Apoptosis induced by cooperation of cytokines is caspase-dependent. A) Caspase activation in HPMC exposed to TNFα/IFNγ for 24 h. Apoptosis was decreased by zVAD (pan-caspase inhibitor), DEVD (caspase-3 inhibitor) and IETD (caspase-8 inhibitor). Note cytokeratin (CK) 8/18 cleavage by caspases as well as the nuclear apoptotic morphology in cells exposed to TNFα/IFNγ (arrowheads). Green: FITC-M30 cytodeath antibody, red: propidium iodide in permeabilized cells. Original magnification ×100. B) Representative diagram of detection of caspase-cleaved CK 8/18 by flow cytometry in HPMC. C) Quantification of caspase-cleaved CK 8/18 by flow cytometry in HPMC treated with TNFα and IFNγ for 24 h. * p<0.005 vs control and # p<0.05 vs TNFα/IFNγ. Mean±SEM of 4 different experiments. D) Flow cytometry diagrams of permeabilized, propidium iodide-stained cells. Note the decrement of hypodiploid cells (arrowhead) in the presence of caspase inhibitors in HPMC. E) Quantification of apoptosis by flow cytometry of DNA content in HPMC exposed to TNFα/IFNγ for 48 h. Apoptosis was decreased by zVAD, DEVD and IETD. *p<0.001 vs control and # p<0.0005 vs TNFα/IFNγ. Mean±SEM of 6 independent experiments.

HPMC apoptosis induced by TNFα and IFNγ was characterized by the appearance of caspase-generated fragments of cytokeratin 8/18 in the cytoplasm and typical morphological features of nuclear apoptosis, such as shrunk, bright and fragmented nuclei (**Fig. 2.A**). Flow cytometry confirmed that TNFα and IFNγ increased the number of cells containing caspase-generated fragments of cytokeratin 8/18 (**Fig. 2.B**) and caspase-3 activity was increased by 1.7±0.4 fold over control at 24 h in a colorimetric assay (p<0.02, not shown). Taken together, these data provide evidence for the involvement of caspases in the process. In order to confirm the requirement for caspases and to explore possible therapeutic strategies, cells were treated with caspase inhibitors. The pan-caspase inhibitor, zVAD, or specific caspase-3 (DEVD) or caspase-8 (IETD) inhibitors prevented morphological features of apoptosis induced by cytokines (**Fig. 2.A**) and the activation of executioner caspases (**Fig. 2.A–C**), and decreased apoptosis as assessed by the presence of hypodiploid cells (**Fig. 2.D, E**). Similar results were obtained in HOMC (not shown). The most effective caspase inhibitor in decreasing the rate of apoptosis, zVAD, was studied in further detail. In mesothelial cells exposed to TNFα and IFNγ, zVAD was unable to prevent eventual cell death (data not shown) despite not increasing the non-apoptotic cytotoxicity of cytokines, as it is the case with other cell types [29]. Caspase inhibition was not *per se* toxic in cells not exposed to cytokine combinations and did not increase the number of dead cells staining with trypan blue (data not shown).

### Impaired wound healing in the presence of a proinflammatory milieu is not rescued by caspase inhibition

**Figure 3:**
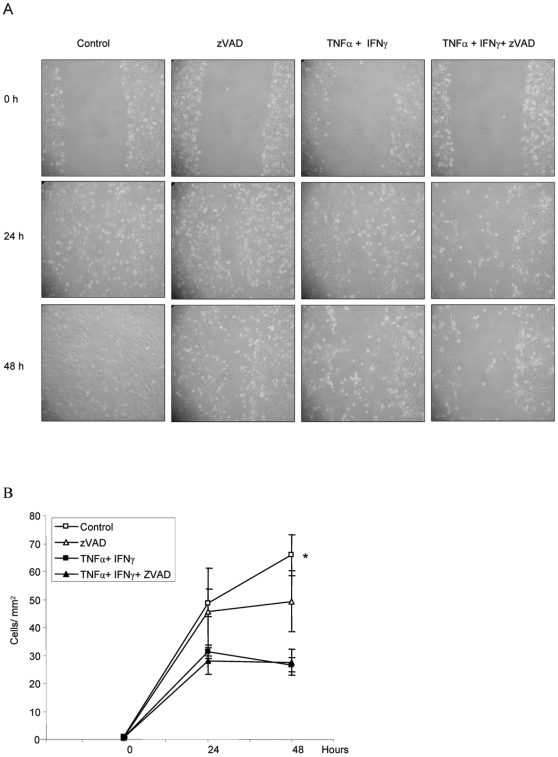
Inflammatory cytokines retard remesothelization. A) Wound healing in HPMC. Contrast phase microscopy. Cells were preincubated with caspase inhibitor or vehicle and cultured in presence of proinflammatory TNFα/IFNγ or control media. Original magnification 20×. B) Quantification of wound healing as number of HPMC cells filling the denuded area (cells/mm^2^). In presence of cytokines there is delayed wound healing that is not improved by zVAD.* p<0.03 control vs. TNFα/IFNγ and control vs TNFα/IFNγ/zVAD. Mean±SEM of 5 different experiments.

Acute demesothelization during peritonitis, unless very severe, is usually a reversible response, and mesothelial cells later repopulate the denuded area. In a wound healing system of HPMC we studied recovery from injury in the absence of exogenous survival factors, aiming to reproduce the adverse microenvironmental conditions of the inflamed peritoneum. Under control conditions, in the absence of proinflammatory cytokines, the mesothelial covering of the wound increased over 48 h (**Fig. 3.A, B**). Caspase inhibition tended to stall the process at late time points. The continuous presence of TNFα and IFNγ prevented wound healing. Inhibition of caspases by zVAD did not rescue wound healing among cells treated TNFα and IFNγ (**Fig. 3.A, B**). IETD and DEVD caspase inhibitors offered no advantage over zVAD (not shown).

### A nanoconjugate Apaf-1 inhibitor prevents cytokine-induced apoptosis and promotes wound healing

**Figure 4:**
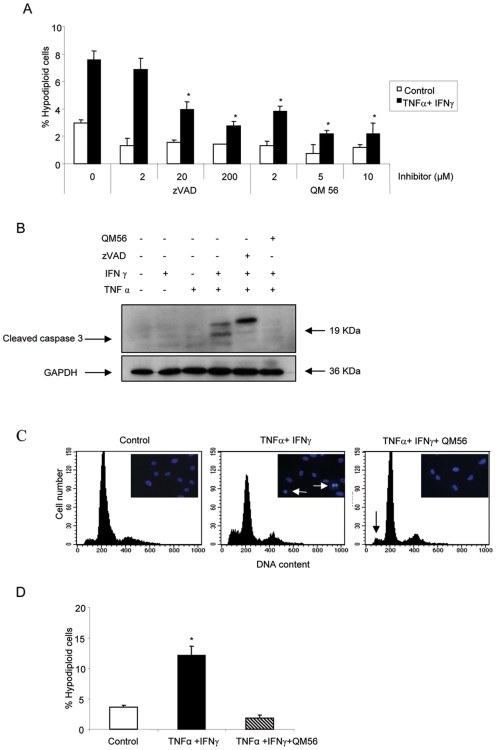
A nanoconjugate Apaf-1 inhibitor (PGA-peptoid QM56) inhibits caspase activation and apoptosis. A) Quantification of apoptosis in HOMC by flow cytometry. HOMC were preincubated with different concentrations of inhibitors or vehicle and cultured with TNFα and IFNγ for 48 hours. Mean±SEM of 3 different experiments *p<0.001vs TNFα/IFNγ. B) QM56 prevents procaspase-3 processing and the appearance of active p17 and p19 caspase-3 fragments 24 h following exposure to TNFα/IFNγ in HOMC. zVAD stalled the process of caspase activation at the level of the p21 precursor as previously described [73], [74]. Binding of zVAD.fmk to this p21 intermediate blocks its activity [74]. Western blot, representative of 3 independent experiments. C) Representative flow cytometry of DNA content diagrams. Cells were preincubated with QM56 and then cultured with TNFα/IFNγ for 48 h. Note the decreased number of hypodiploid apoptotic HPMC in TNFα/IFNγ/QM56 treated cells (black arrow). Inset: nuclear apoptotic morphology in cells exposed to TNFα/IFNγ and stained with DAPI (white arrows). D) Quantification of apoptosis by flow cytometry of DNA content in HPMC. Mean±SEM of 6 different experiments. *p<0.002 vs cells treated with TNFα/IFNγ/QM56.

**Figure 5:**
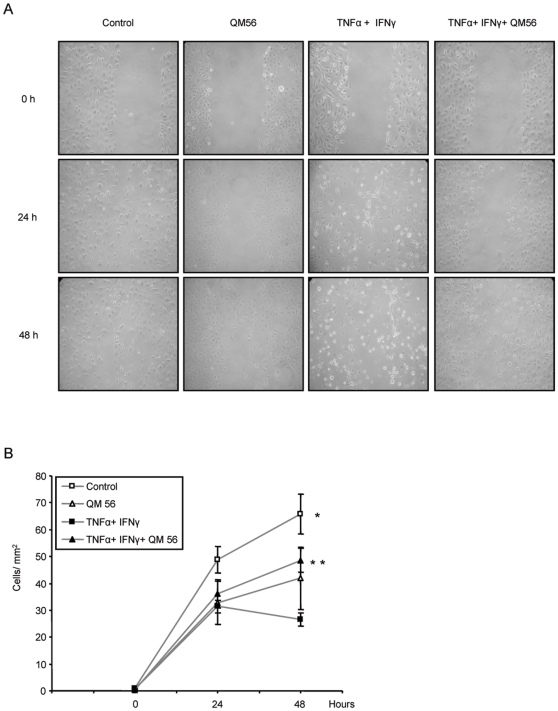
QM56 preserves remesothelization. A) Wound healing in HPMC. Contrast phase microscopy. Cells were preincubated with Apaf-1 inhibitor and cultured in presence of proinflammatory medium (TNFα and IFNγ). Original magnification 20×. B) Quantification of wound healing in HPMC as number of cells filling the denuded area (cells/mm^2^). The delayed wound healing induced by TNFα/IFNγ is recovered in presence of QM56. * p<0.03 control vs. TNFα/IFNγ ** p<0.003 TNFα/IFNγ/QM56 vs. TNFα/IFNγ. Mean±SEM of 5 different experiments.

Formation of the apoptosome is a key event in the apoptotic signaling pathway. First-in-class polyglutamic acid (PGA)-based Apaf-1 inhibitor nanoconjugates (PGA-peptoids) are well suited for in vivo applications [30]–[32]. The PGA-peptoid QM56 prevented, in a dose-dependent manner, mesothelial cell apoptosis induced by a combination of cytokines, as shown for HOMC (**Fig. 4.A**). The QM56 concentration chosen for the rest of the experiments (10 µM) had an antiapoptotic activity similar to 200 µM zVAD (**Fig. 4.A**). In cultured mesothelial cells QM56 prevented caspase-3 processing induced by cytokines, as shown for HOMC (**Fig. 4.B**) and completely prevented the increase in caspase 3 activity induced by TNFα/IFNγ in these cells (TNFα/IFNγ 1,7±0.4 vs TNFα/IFNγ/QM56 0,56±0.18 fold over control, p<0.03, not shown). Similar to observations with caspase inhibitors, QM56 prevented apoptosis induced by TNFα and IFNγ in mesothelial cells, as shown for HPMC (**Fig. 4.C,D**). QM56 did not modify the percentage of cells in the S+M phases of the cell cycle (not shown). By contrast to classical caspase inhibitors, QM56 restored the wound healing capacity of mesothelial cells in the presence of cytokines, as shown for HPMC (**Fig. 5.A, B**).

### Apaf-1 inhibition, but not caspase inhibition, improves long-term recovery from inflammatory injury

**Figure 6:**
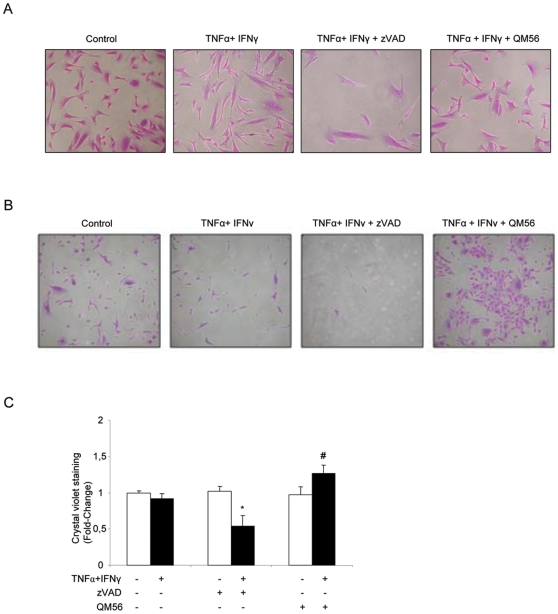
Caspase inhibition compromises long-term recovery from inflammatory injury while Apaf-1 inhibition does not. A) HPMC plated on twelve-well plates were preincubated with inhibitors or vehicle and exposed to TNFα/IFNγ for 24 h. Cells were then trypsinized, washed, and seeded in Petri dishes in the presence of complete medium with 20% FCS for 5 days, but without cytokines or inhibitors, to allow for their recovery. Cells were stained with crystal violet after 5 days. Representative of 3 different experiments. B) Similar results were obtained with HOMC. HOMC were preincubated with inhibitors or vehicle and exposed to TNFα/IFNγ for 48 h. Then the cells were trypsinized, washed and seeded without inhibitors and cytokines for 5 days. Representative of 4 different experiments. C) Quantification of crystal violet staining in HOMC. Mean±SEM of 4 different experiments. *p<0.03 vs TNFα/IFNγ; #p<0.02 vs TNFα/IFNγ.

We next explored the potential long-term consequences of caspase inhibition in the context of transient, short-term inflammation. Cells were cultured for 24 to 48 h in the presence of TNFα and IFNγ with or without inhibitors and then trypsinized, seeded in new plaques and left to recover for 5 days in the presence of survival factors from serum but in the absence of cytokines or inhibitors. This mimics intraperitoneal events during the recovery from human peritonitis with an adequate response to antibiotics. Despite the acute increase in cell death and delayed wound healing observed in the presence of TNFα and IFNγ, the removal of these cytokines from the media and the addition of exogenous survival factors was associated with a degree of recovery close to that obtained in control HPMC (**Fig. 6.A**). The initial presence of the cytokine combination and the pan-caspase inhibitor led to a reduced final cell number, suggesting loss of regenerative potential (**Fig. 6.A**). Similar results were observed in HOMC from non-dialysis patients (**Fig. 6.B,C**). Short-term exposure of control cells to zVAD did not impair long-term recovery, indicating that this is not an intrinsic toxic effect of zVAD (**Fig. 6.C**).

By contrast to caspase inhibition, QM56 did not impair the long-term regeneration of HPMC or HOMC subjected to an inflammatory milieu (**Fig. 6A–C**). Indeed, QM56 significantly improved recovery (**Fig. 6.C**).

### Apaf-1 inhibitor protects from inflammation-induced apoptosis in vivo

**Figure 7:**
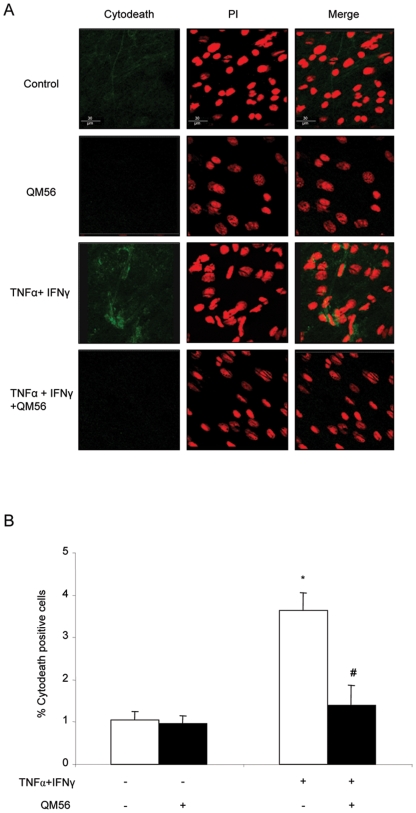
QM56 prevents mesothelial cell apoptosis induced by intraperitoneal cytokine administration in vivo. Mice were injected ip with 250 ng/mL TNFα and 300 U/mL IFNγ at time 0 and sacrificed at 48 h. QM56 or vehicle was administrated 1 h before the cytokines and 5 h later. A) Representative images of Cytodeath (green)/propidium iodide co-staining. Note an increased number of cells with green cytoplasm indicative of caspase-mediated apoptosis in the sample from the mouse injected with TNFα/IFNγ. Magnification ×120 B) Quantification of positive cells (green) using Image Pro plus software in 5 fields per mouse (around 600 cells). Mean±SEM of 5 mice per group. * p<0.001 vs. control: # p<0.001vs.TNFα IFNγ.

**Figure 8:**
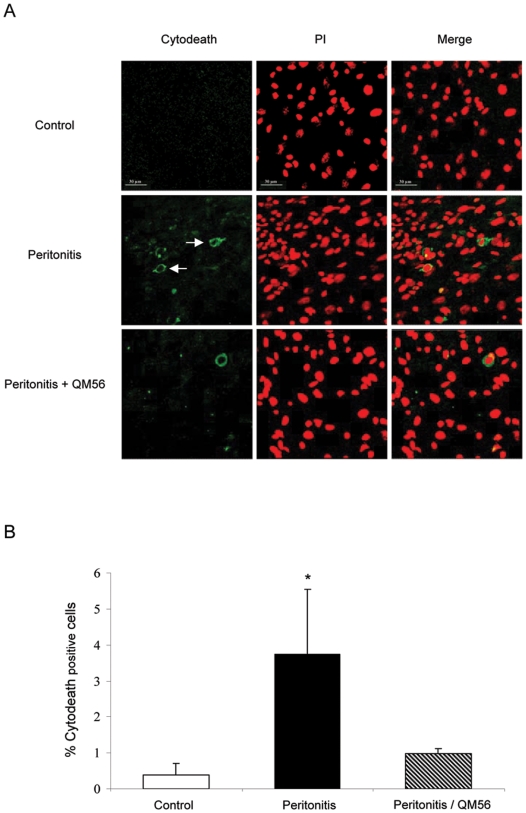
QM56 prevents mesothelial cell apoptosis during *S. aureus* peritonitis in mice. Mice were injected ip. with 5×10^8^ c.f.u. *S. aureus* at time 0 and sacrificed at 48 h. QM56 or vehicle was administrated 1 h before the *S. aureus* and 5 h later A) Representative images of Cytodeath (green)/propidium iodide co-staining. Note an increased number of cells with green cytoplasm (arrows) indicative of caspase-mediated apoptosis in the sample from the mouse injected with *S. aureus*. Apoptosis was prevented by QM56 Magnification ×120. B) Quantification of cytodeath positive cells (green) using Image Pro plus software in 5 fields per mouse (around 600 cells). Mean±SEM of 5 mice per group. * p<0.05 vs control.

We next explored the potential of QM56 to modulate cell injury in vivo. Cytokeratin fragmentation, a marker of apoptosis that precedes nuclear changes [7], was chosen because in vivo early apoptotic cells are rapidly expelled from cell monolayers and engulfed by professional phagocytes [35]. In mice the intraperitoneal administration of TNFα and IFNγ resulted in an increased rate of mesothelial cell apoptosis at 48 h (**Fig. 7**). *S. aureus* peritonitis also resulted in mesothelial cell apoptosis (**Fig. 8**). The Apaf-1 inhibitor QM56 prevented mesothelial cells apoptosis induced by both inflammatory milieus in vivo (**Fig. 7** **,** **8**).

## Discussion

We chose mesothelial cell injury as a model for inflammation-induced tissue injury because mesothelial cell loss is a clinical problem in PD. In addition, the easy access to the peritoneal cavity allows the local delivery of therapeutic agents. We now report that in inflammation-induced mesothelial cell injury cytokines cooperate to induce apoptosis in primary cultures of mesothelial cells obtained from either PD patients or non-uremic individuals. We have further explored possible therapeutic strategies to prevent cell injury and have identified Apaf-1 inhibition as a novel approach that prevents acute inflammation-induced mesothelial cell injury and allows recovery.

Mesothelial cells are lost by apoptosis in the course of human bacterial PD peritonitis [5]–[7]. We now observed an increased mesothelial apoptosis rate in an experimental model of bacterial peritonitis. In the course of peritonitis multiple inflammatory mediators are released locally, including IFNγ and TNFα [33], [34], [36], [37]. Interestingly, the highest peritoneal IFNγ and TNFα levels are found in peritonitis caused by highly virulent microorganisms, such as *S. aureus*, precisely the ones that cause more severe demesothelization and that may lead to irreversible peritoneal injury after a single, severe peritonitis episode [33], [34]. Since multiple inputs are perceived by cells in a proinflammatory milieu, it is relevant to study the actions of cytokine combinations [12]. Our studies show that a TNFα/IFNγ combination promoted apoptosis of cultured mesothelial cells, and explore novel therapeutic approaches to prevent inflammation-induced tissue injury that do not target TNFα or IFNγ. Both TNFα and IFNγ are required for an effective antibacterial defense. TNFα antagonists have been marred by an increased rate of severe infections [38]. This is more obvious with infliximab, which also inhibits IFNγ secretion [38] [39]. This suggests that during infection rather than antagonize cytokines, we should strive for the selective therapeutic manipulation of their specific adverse effects that promote tissue injury, such as parenchymal cell apoptosis.

A single episode of very severe peritonitis may cause irreversible demesothelization and peritoneal fibrosis. However, more often peritonitis is mild to severe, and is followed by partial or total recovery of mesothelial integrity. Both the magnitude of the initial acute loss of mesothelium and the ability of cells to regenerate are important factors in the recovery phase. Our experimental design has addressed how therapeutic agents modulate the initial lethal response to inflammation, the subsequent ability of the mesothelium to repopulate a demesothelized area in the presence of ongoing adverse microenvironmental conditions, and the later recovery phase taking place under a milder microenvironment. The continuous presence of inflammatory cytokines increased the rate of apoptosis and impaired regeneration of the mesothelial layer. However, restoration of the normal cell milieu, with disappearance of inflammatory cytokines and the renewed presence of survival factors, lead to recovery. We now show that cytokine-mediated mesothelial cell apoptosis depends on caspase activation and is prevented by caspase inhibition. However, caspase inhibition did not allow mesothelial layer regeneration in the presence of cytokines to proceed, and, more ominously, severely impaired the long-term regeneration of the mesothelium once cytokines were removed. There are several possible explanations for this phenomenon. First, massive induction of necrosis may occur in cells exposed to members of the TNF superfamily when caspases are inhibited [12], [29], [40]. In this regard, caspase inhibitors did not transform a moderate degree of mesothelial cell apoptosis into a catastrophic rate of necrosis, as zVAD/TNFα/IFNγ did not increase the percentage of necrotic cells over TNFα/IFNγ. However, caspase inhibition may not prevent eventual non-apoptotic death in cells rescued from apoptosis. Second, caspase inhibitors may inhibit non-apoptotic roles of caspases [41]. Cell migration and mitosis are required to repair a cell monolayer wound [42]. Recently a spate of non-apoptotic caspase actions on cell proliferation and migration have been described that favor the recovery process [26]–[28], [43], [44]. As an example, TNFα is deleterious for the liver in the course of inflammation, through induction of apoptosis [45], [46]. However, liver regeneration following partial hepatectomy requires TNFα signaling through the TNFR-1 receptor and caspase-8 activation that primes hepatocytes to respond to mitogens [26], [47], [48]. Caspase-3 and caspase- 11 may promote cell migration [27], [28].

Chemical inhibition of Apaf-1 by a nanoconjugate molecule [30], [31], [49] prevented mesothelial cell apoptosis induced by the combination of cytokines in cell culture and in vivo, suggesting that Apaf-1 is required for mesothelial cell death to occur. This suggests that mitochondria are engaged during TNF-induced mesothelial cell apoptosis and, thus, that mesothelial cells should be considered type II with regard to their response to death receptor activation [50]–[53]. Following release of cytochrome c from mitochondria, Apaf-1 is required for caspase-9 activation, setting in motion a rapid amplification of the death signal through activation of caspase-3 and downstream caspases leading to further mitochondrial injury [54]–[56]. Contrary to caspase inhibitors, the inhibitor of Apaf-1 also restored the wound healing capacity and promoted long-term recovery of mesothelium. Two, non-mutually exclusive hypothesis, might explain the differences observed between caspase and Apaf-1 inhibitors. In one of them additional functions of caspases, resulting from caspase activation not mediated by proapoptotic stimuli, and, thus, not targeted by inhibition of Apaf-1, may explain the differences. In the previous paragraph we mentioned several non-apoptotic functions of caspases that may not be targeted by the Apaf-1 inhibitor. In addition, as an example, in the absence of cellular stress human glioblastoma cells exhibit a constitutive activation of caspases *in vivo* and *in vitro*. Basal caspase 3 and caspase 8 activity promotes migration and invasiveness in glioblastoma cells and inhibition of caspases decreases the migration and the invasiveness of cells [57]. The administration of low doses of caspase inhibitors may block glioma cell motility without affecting the execution of apoptotic cell death [57]. In the second hypothesis, additional functions of Apaf-1 inhibitors, unrelated to caspases, may underlie this observation. For example, inhibition of the recently described cell cycle arrest induced by Apaf-1 [58]. Although the role of Apaf-1 in the DNA damage checkpoint may raise concerns on the carcinogenicity of Apaf-1 targeting, life-long lack of Apaf-1 in Apaf-1-/- mice has not been reported to result in an increased incidence of tumors [59], [60]. Additional actions of the Apaf-1 inhibitor cannot be excluded, since a related compound, in addition to inhibiting both functions of Apaf-1, also protects mitochondria [49]. The therapeutic potential of the Apaf-1 inhibitor was confirmed in vivo, where it prevented mesothelial cell loss induced by either TNFα/IFNγ or by bacterial peritonitis. Certain stimuli, such as osmotic stress and Staphylococcus aureus promote mesothelial cell death with features of apoptosis that is no prevented by caspase inhibitors [16], [18]. It will be interesting to test the efficacy of Apaf-1 inhibitors in preventing cell death in these models.

In this paper we have focused on the monomicrobial peritonitis characteristic of PD as a proof-of-concept model. In humans these peritonitis are routinely treated by the intraperitoneal administration of therapeutic agents, as used in our models. Polymicrobial peritonitis resulting from visceral perforation, as exemplified by the cecal ligation and puncture model is also a clinically relevant model [61]. However, intravenous therapy is used in humans with perforated bowels, and thus would be the most relevant rout to be studied in animals. This route presents currently unresolved pharmacokinetic issues with the Apaf-1 inhibitor we used. Apaf-1 inhibitors should also be tested in this model in future studies.

In summary, our data indicate that a chemical compound targeting Apaf-1 prevented inflammation-induced tissue damage, exemplified by the peritoneum, without the adverse consequences of approaches targeting caspases. Our data offer a word of caution when considering the use of caspase inhibitors in the clinical setting: the potential benefits obtained by limiting the initial apoptosis wave may be offset by an impaired recovery from parenchymal cell injury.

## Materials and Methods

### Mesothelial cell cultures

The study was approved by the clinical ethics committee of Fundación Jiménez Díaz and written informed consent was obtained. Human peritoneal mesothelial cell (HPMC) were cultured from peritoneal effluents from 6 stable CAPD patients as previously described [6], [62]. Human omental mesothelial cells (HOMC) were obtained from omentum from 6 non-PD patients who underwent unrelated elective abdominal surgery [63], [64].

### Reagents

Human IFNγ and TNFα (Peprotech, London, UK) were used at concentrations based on prior experience with other cell types [22], [65] and expected to occur in vivo during peritonitis, specially in patients infected with highly virulent bacteria such as *S. aureus*, adjusted for PD fluid dilution [33], [34]. Peritoneal effluent concentrations of 4.5 ng/ml TNFα and 45 U/ml IFNγ have been reported in the effluents of PD patients [33], [34]. Higher cytokine concentrations may be reached locally in mesothelial cells in close contact with paracrine cytokine-secreting leukocytes, as well as in non-PD peritonitis, which lacks the diluting effect of PD solutions. The combination of 100 U/mL (5 ng/mL) TNFα and 20 U/mL IFNγ already increased significantly the rate of apoptosis in HOMC by 1.5 fold, but the effect increased with dose up of 250 U/mL TNFα and 300 U/mL IFNγ, which were used if not otherwise specified. The antiapoptotic polymeric nanomedicine, PGA-peptoid QM56, is the result of the conjugation of a novel Apaf-1 inhibitor (peptoid 1) to poly-L-glutamic acid (PGA) [30], [31]. The initial dose range was chosen based on published dose-response curves and dose-response studies in human mesothelial cells induced to undergo apoptosis by exposure to staurosporin (Figure S1) [30], [31]. Additional dose-response studies were performed in HOMC exposed to IFNγ and TNFα and the concentration chosen for the rest of the studies was 10 µM. The dose of caspase inhibitors was based on previous dose-response studies [66], [67]. In addition dose-response studies were performed in HOMC exposed to IFNγ and TNFα and the concentration chosen for the rest of the studies was 200 µM zVAD.

### Studies of cell death and cleavage by caspases

Mesothelial cells were cultured to subconfluence in twelve-well plates and rested in serum-free media for 24 h. Then, IFNγ and/or TNFα were added. Cells were preincubated with apoptosis inhibitors for 1 h. [24], [68].

Apoptosis was assessed by functional and morphological studies. Cell DNA content was quantified by flow cytometry in permeabilized, propidium iodide-stained cells [22], [65]. Permeabilization allows entry of propidium iodide in all cells, dead and alive. Apoptotic cells are characterized by a lower DNA content (hypodiploid cells) because of nuclear fragmentation. Caspases are key effectors of apoptosis. Evidence of caspase activation was assessed studying caspase processing by Western blot. In addition, flow cytometry or microscopy was used to quantify the appearance of a specific epitope generated by caspase cleavage of cytokeratin 18 and identified by the M30 cytodeath antibody (Roche Biochemicals, Mannheim, Germany). This epitope is generated only in epithelial and mesothelial cells, not in leukocytes, and is not present in native cytokeratin 18 [7], [69].

For morphologic assessment of apoptosis, cells were cultured in chamber slides (Labtek, Nunc, Naperville, IL), fixed with methanol:acetone (1∶1), and stained with DAPI [65], or FITC-M30 cytodeath (1∶250 Roche) and propidium iodide. Thus, the typical condensed, shrunk and fragmented nuclei of apoptotic cells were identified

Trypan blue staining of freshly collected cells was used to assess cell death due to either apoptosis or necrosis [29].

### Caspase-3 activity

HOMC were preincubated with Apaf-1 inhibitor or vehicle and cultured with IFNγ and TNFα for 24 hours. Caspase-3 activity (MBL, Japan) was measured following the manufacturer's instructions. In brief, cell extracts (100 µg of protein) were incubated in half-area 96-well plates at 37°C with 200 µM DEVD-pNA peptide in a total volume of 100 µl. The pNA light emission was quantified using a spectrophotometer plate reader at 405 nm. Comparison of the absorbance of pNA from an apoptotic sample with an uninduced control allows determination of the fold increase in caspase activity [70].

### Wound healing

The wound-healing model was modified from Yung et al. [71]. Mesothelial cells were cultured to confluence in twelve-well plates and rested in serum-free media for 24 h. The monolayer was injured with a sterile pipette tip and IFNγ, TNFα and apoptosis inhibitors were added to serum-free media. Remesothelization was followed for up to 48 h by marking the injured area and counting cells inside it at different time points.

### Long-term recovery

Long-term recovery was assessed by a modification of the colony-forming assay. Cells in twelve-well plates were exposed to cytokines and apoptosis inhibitor or vehicle for 24-48 h in serum-free media and then trypsinized, washed, and seeded in Petri dishes in the presence of complete medium with 20% FCS and without cytokines or inhibitors of apoptosis. Cell number was estimated at 5 days by crystal violet staining, absorbance was measured at 550 nm [72].

### Animal model

C57BL/6 mice, 3 month-old, were injected ip with a single dose of 300 U/mL IFNγ and 250 ng/mL TNFα, or 0.5 ml PBS. QM56 (10 µM drug-equiv.) or vehicle (0.5 ml PBS) were administered 1 h before and 5 h later (total 4 groups, 5 mice per group). Mice were sacrificed at 48 h. The dose and timing were based on cell culture results.

*Staphylococcus aureus* ATCC 25923 (American Type Culture Collection, Manassas, VA, USA) was used to induce peritonitis. The experimental protocol was described previously [25] C57BL/6 mice, 3 month-old were injected i.p. with 5×10^8^ colony forming units (c.f.u.) *S. aureus* in 1 mL PBS or with PBS. At 48 hours mice were sacrificed. Peritonitis was mild and animals had spontaneously cleared *S. aureus* by 48 h.

In both models mesenteric windows were placed on glass slides and stained with Cytodeath and propidium iodide. Studies were conducted in accord with the NIH Guide for the Care and Use of Laboratory Animals.

### Statistics

Statistical analysis was performed using SPSS 11.0 statistical software. Results are expressed as mean±SEM. Significance at the p<0.05 level was assessed by Student's t test and one- way ANOVA with Bonferroní's correction or Mann-Whitney and Kruskal-Wallis tests.

## Supporting Information

Figure S1 Quantification of apoptosis in HPMC by flow cytometryof DNA content. HPMC were preincubated with QM56 for 1 hour and cultured with 100 nM staurosporin(STS) for 24 hours. * p<0.001 vscontrol (co) or different concentrations of inhibitor. Mean±semof 4 different experiments.
(0.33 MB TIF)
