# Supplementary figures and images for: A Nanoconjugate Apaf-1 Inhibitor Protects Mesothelial Cells from Cytokine-Induced Injury
Source: PLoS One. 2009 Aug 13;4(8):e6634. doi: 10.1371/journal.pone.0006634 (PMC2722088; doi:10.1371/journal.pone.0006634)

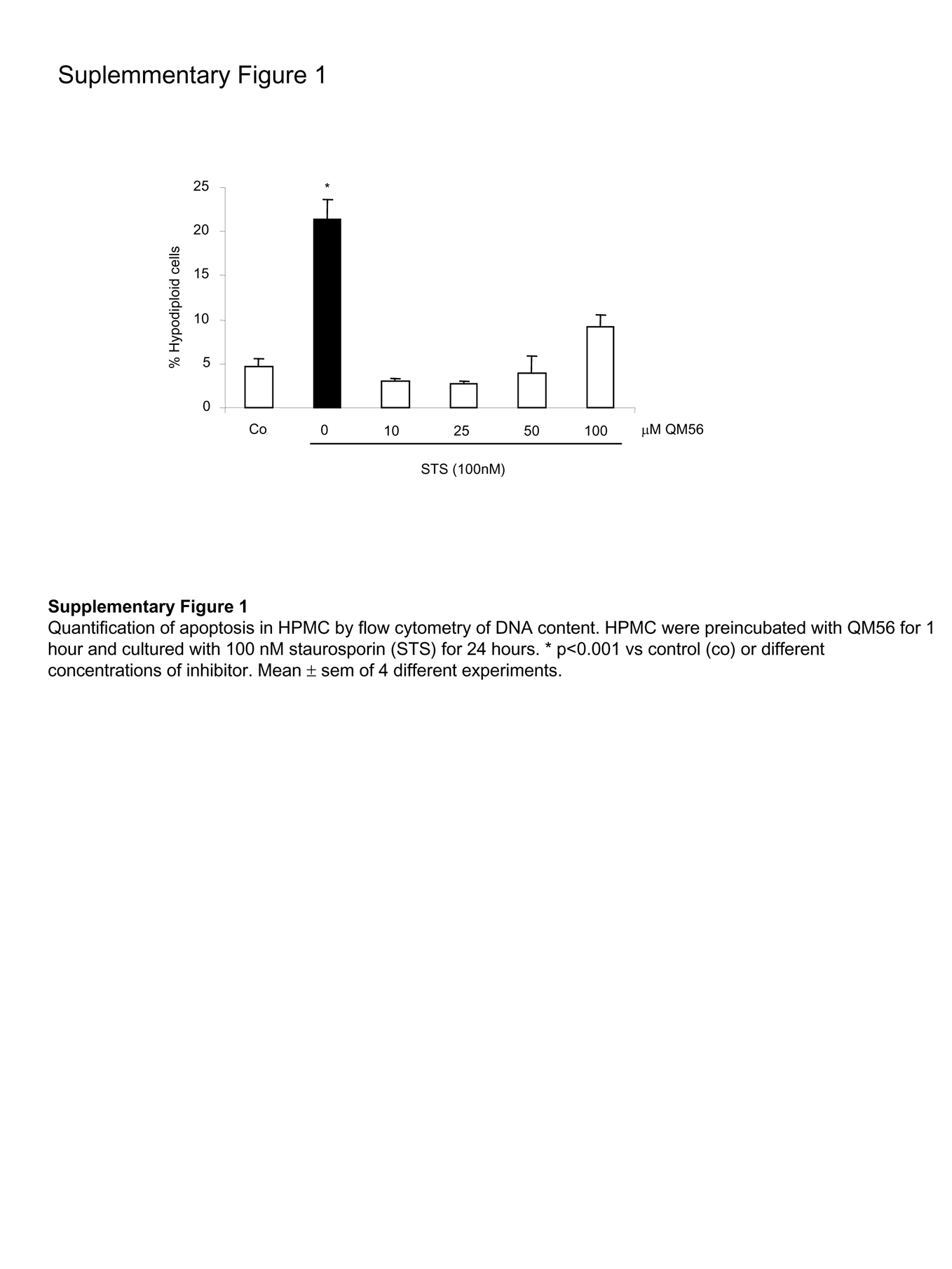

Supplement: Figure S1 — Quantification of apoptosis in HPMC by flow cytometryof DNA content. HPMC were preincubated with QM56 for 1 hour and cultured with 100 nM staurosporin(STS) for 24 hours. * p<0.001 vscontrol (co) or different concentrations of inhibitor. Mean±semof 4 different experiments. (0.33 MB TIF) [file pone.0006634.s001.tif]
